# Character strengths, psychological distress, anxiety, and depression in adolescents and young adults with gynecologic cancer: A network analysis

**DOI:** 10.1016/j.apjon.2026.101027

**Published:** 2026-09-10

**Authors:** Ning Qin, Yuxuan Li, Huiyi Zhang, Juan Luo, Dongren Qin, Jie Zhu, Jian Peng, Zitong Lu, Jianfei Xie, Shuijing Yi, Peter H.F. Ng

**Affiliations:** a Nursing Department, The Third Xiangya Hospital, Central South University, Changsha, Hunan Province, China; b Xiangya School of Nursing, Central South University, Changsha, Hunan Province, China; c Department of Gynecology, The Third Xiangya Hospital, Central South University, Changsha, Hunan Province, China; d Department of Computing and Department of Rehabilitation Science, The Hong Kong Polytechnic University, Hong Kong SAR, China

**Keywords:** Psychological distress, Character strengths, Adolescent, Young adult, Genital neoplasms, Network analysis

## Abstract

**Objective:**

Adolescents and young adults with gynecologic cancer face distinctive psychosocial challenges. We examined associations between character strengths and psychological distress, anxiety, and depression and identified candidate strength-related nodes for future psychosocial research.

**Methods:**

This cross-sectional study included 271 participants diagnosed with gynecologic cancer during adolescence or young adulthood. Gaussian graphical models estimated conditional associations and central and bridge nodes. Bayesian networks explored directed probabilistic dependencies under theory-constrained and unconstrained specifications. In silico simulations estimated model-implied outcome changes following hypothetical 1-standard deviation (SD) increases in individual character strengths.

**Results:**

The prespecified character-strength and psychological-outcome communities showed dense within-community and sparse between-community connections. Leadership, Creativity, Curiosity, and Teamwork had the highest strength centrality among character strengths. Psychological distress had the highest bridge strength among psychological-outcome nodes (0.148); Curiosity and Zest had the highest bridge strengths among character-strength nodes (both = 0.112). Results were similar after covariate adjustment. The theory-constrained and unconstrained Bayesian networks each retained 31 directed edges, 28 of which overlapped. The Curiosity–depression dependency was retained in both models, whereas the orientation of the Zest–distress dependency varied by model specification. A hypothetical 1-SD increase in Curiosity corresponded to the largest model-implied decrease in overall outcome severity (−0.230 SD), including depression (−0.306 SD); the model-implied change for Zest was primarily observed for distress (−0.123 SD).

**Conclusions:**

Curiosity and Zest were candidate strength-related nodes with distinct, model-dependent associations with psychological outcomes. These hypothesis-generating findings require confirmation in longitudinal and intervention studies before clinical application.

## Introduction

Gynecologic cancers (GC), including malignancies of the cervix, ovary, uterus, vagina, and vulva, represent a significant health challenge for women.^1^ While these malignancies are frequently diagnosed in older populations, they also critically impact a distinct demographic defined by the National Comprehensive Cancer Network (NCCN) as adolescents and young adults (AYAs)—individuals diagnosed between the ages of 15 and 39 years.^2^ The global cancer burden within this age group is substantial and increasing; in 2022, there were an estimated 1.3 million new cancer cases and 380,000 deaths among AYAs worldwide, with projections indicating a further rise by 2050.^3^ Within this expanding demographic, adolescents and young adults with gynecologic cancer (AYGC) represent a unique subgroup facing complex challenges. Compared with older patients, adolescents and young adults with gynecologic cancer may experience a double burden where the trauma of a cancer diagnosis and the rigors of treatment—such as fertility-threatening surgery, hormonal disruption, and changes in body image—intersect with critical developmental milestones such as education, career establishment, intimate relationships, and family planning.^4^^,^^5^ Consequently, this group is particularly vulnerable to adverse psychosocial outcomes,^6^ underscoring the urgent need for dedicated clinical and research attention to AYGC.

Psychological distress is a broad, multidimensional unpleasant experience in psycho-oncology, encompassing emotional, social, physical, practical, and existential concerns.^7^ Anxiety and depression are among the most common and clinically meaningful emotion-related manifestations within or alongside this broader distress experience. Anxiety is particularly important in AYGC because it is closely tied to anticipatory threat and uncertainty, including concerns about prognosis, recurrence, treatment-related adverse effects, fertility preservation, potential loss of ovarian or uterine function, body-image changes, intimate relationships, and future family roles.^8^ Depression may be more closely associated with experiences of loss, functional impairment, and persistent emotional burden, whereas psychological distress reflects the broader subjective burden of the illness experience.^8^^,^^9^ Therefore, examining psychological distress, anxiety, and depression as separate but closely related nodes may help clarify different domains of cancer-related psychological burden. Although psychosocial interventions have been developed for people with cancer, their effects in AYGC remain variable and sometimes inconclusive.^10^^,^^11^ One possible explanation is that current approaches often treat psychological distress as a homogeneous construct, relying on aggregate scores that may obscure heterogeneity in patients’ emotional and illness-related experiences.^12^ For instance, the processes underlying fertility-related worry may differ from those underlying fatigue-related depressive symptoms, yet traditional latent variable approaches may conflate these distinct experiences.^13^ A network perspective may therefore help identify central and bridge nodes within the broader psychological burden network, thereby providing a basis for examining how these symptoms are linked with adaptive personal resources.^14^

In contrast to the traditional deficit-based model of psychopathology, positive psychology offers a complementary perspective by focusing on adaptive personal resources, particularly character strengths (CS).^15^ Defined as positive personality traits that reflect universal virtues, such as curiosity, zest, self-regulation, kindness, and teamwork, CS are important internal resources that may support adaptation to adversity.^16^ Research indicates that patients with cancer who use specific CS demonstrate greater post-traumatic growth and lower levels of psychopathology.^17^ For AYGC, who are in a dynamic stage of personality development, these strengths may be particularly relevant to coping with the psychological impact of a cancer diagnosis. However, most existing studies have examined only linear relationships between total CS scores and overall distress, overlooking how specific strengths may be differentially associated with specific psychological outcomes.^18^^,^^19^

Emerging evidence suggests that specific personal attributes and self-regulatory mechanisms play important roles in shaping psychological adaptation to cancer.^20^ Duan et al.^19^ noted that young patients with cancer who had lower self-control also had significantly higher levels of psychological distress. Similarly, in a large-scale survey of 1248 patients across multiple cancer types, Langford et al.^21^ found that personality profiles characterized by high neuroticism and low conscientiousness—traits closely linked to self-control deficits—were significantly associated with a heavier symptom burden. However, associations between CS and cancer-related psychological burden are likely to be more complex than these linear findings suggest. In the Chinese context, several sociocultural and healthcare-related factors may be particularly relevant for AYGC, including family-centered decision making, expectations regarding marriage and childbearing, stigma associated with reproductive cancers, cultural norms around emotional restraint and non-disclosure, and uneven access to professional psychosocial support.^22^^,^^23^ These contextual factors may be related to both how patients experience and disclose psychological distress and how they mobilize personal strengths during illness adjustment. Given the inconsistent evidence regarding which strengths are most closely associated with psychological outcomes, and the limited attention to context-specific strength–symptom interdependencies,^24^ it is important to examine conditional associations among CS, psychological distress, and emotion-related symptoms in Chinese AYGC.

Conceptually, CS can be viewed as relatively stable positive psychological traits or resources, whereas psychological distress, anxiety, and depression represent more state-like psychological outcomes in the current cancer context. Longitudinal evidence suggests that character-related strengths may be prospectively associated with better mental health and lower odds of depression,^25^ while prolonged or chronic psychological distress may, in turn, be associated with changes in personality traits.^26^ Therefore, this study was guided by the theoretical assumption that CS may be associated with psychological outcomes, but this assumption was treated as a theory-informed framework rather than as evidence of causal direction. Accordingly, this study used an integrated, cross-sectional network analytic framework combining Gaussian graphical models, Bayesian network analysis, and in silico simulation analysis to examine associations among CS, psychological distress, anxiety, and depression in Chinese AYGC. Gaussian graphical models were used to estimate conditional associations and identify central and bridge nodes; Bayesian network analysis was used exploratorily to examine potential directed probabilistic dependencies under specified model assumptions; and in silico simulation analysis was used to generate hypotheses regarding candidate strength-based nodes for future research. This integrated approach may contribute to a more nuanced understanding of strength–symptom interrelationships and provide preliminary directions for future longitudinal and intervention studies.

## Methods

### Study design and participants

This cross-sectional study was conducted between July 2024 and April 2025 in the gynecologic oncology departments of two tertiary hospitals in Hunan Province, China. The study was designed and reported in accordance with the Strengthening the Reporting of Observational Studies in Epidemiology (STROBE) statement.^27^ AYAs who had been diagnosed with histologically confirmed gynecologic cancer between the ages of 15 and 39 years were recruited. Eligible cancer types included cervical, endometrial, ovarian, vaginal, and vulvar cancer.^2^ Eligible participants were aware of their diagnosis, had sufficient literacy and communication skills, and voluntarily agreed to participate. Participants were excluded if they had a history of psychiatric disorders, severe comorbidities, cognitive impairment, or substance dependence.

### Sample size

Sample size planning was informed by methodological considerations for psychological network analysis, particularly the need to evaluate network accuracy and stability after estimation, as discussed by Epskamp et al.^28^ Because this study used exploratory network models rather than a conventional hypothesis-testing design, the sample size was not determined by a single formal power-calculation procedure based on effect size, alpha level, power, or allocation ratio. Instead, it was planned pragmatically with reference to the number of nodes, expected network complexity, and recruitment feasibility, and was subsequently evaluated using post-estimation stability analyses.

First, the planned network comprised 18 nodes, including 15 CS indicators, psychological distress, anxiety, and depression. For descriptive transparency, k (k−1)/2 represents the number of possible undirected edges in a k-node network; thus, the present 18-node network contained 18 × 17/2 = 153 possible edges. If node-related parameters are additionally counted, k (k−1)/2 + k yields 171 parameters. This parameter count was used as a planning reference rather than as a formal sample size requirement. Applying an anticipated invalid-response allowance of approximately 20% to this planning reference yielded a recruitment target of approximately 214 participants. Second, expected network complexity was considered because the model included a relatively large CS community, a psychological-outcomes community, and potential cross-community bridge associations. To reduce overfitting and improve interpretability, the primary Gaussian graphical model (GGM) was estimated using regularized partial correlations with extended Bayesian information criterion model selection. Third, recruitment feasibility was considered because AYGC is a relatively specific clinical population; therefore, eligible participants identified in the two participating tertiary hospitals during the study period were approached where feasible. Finally, Bayesian network analysis was conducted as a supplementary exploratory method to examine potential directed probabilistic dependencies under model assumptions, with bootstrap-based edge retention used to quantify how often edges were recovered within the specified procedure rather than to establish causal direction. Ultimately, 288 questionnaires were distributed, and 17 were excluded because of participant withdrawal, substantial incompleteness, or logical inconsistencies. A total of 271 valid questionnaires were included in the final analysis, yielding an effective response rate of 94.10%. Post-estimation uncertainty and stability were assessed using edge-weight accuracy and centrality stability analyses for the GGM and bootstrap-based edge retention for the Bayesian network.

### Data collection procedure and quality control

Data were collected by four trained research assistants. Before data collection, potentially eligible adolescent and young adult patients with gynecologic cancer were preliminarily identified through the hospital information system of the participating wards. The research assistants then verified eligibility according to the predefined inclusion and exclusion criteria. For eligible participants, the research assistants explained the study purpose, survey procedure, voluntary nature of participation, confidentiality of data, and relevant precautions. After electronic informed consent had been obtained, participants completed the questionnaire package in electronic format.

To ensure procedural consistency, all four research assistants received standardized training before data collection. The training covered the study purpose, inclusion and exclusion criteria, informed consent procedures, principles for questionnaire guidance, and requirements for data checking. During questionnaire completion, if participants had difficulty understanding an item or responding independently, the research assistants provided standardized clarification and necessary assistance without giving any leading explanations or influencing participants’ responses. The electronic questionnaire was configured with mandatory response fields to reduce missing data. After completion, the research assistants checked each submitted questionnaire on site for completeness and logical consistency. Questionnaires with participant withdrawal, substantial incompleteness, or obvious logical inconsistencies were excluded from the final analysis.

### Measures

#### Demographic and clinical characteristics

A structured questionnaire, informed by prior research,^29^ was used to collect sociodemographic data, including education, occupation, economic satisfaction, residence, and family relationships. Clinical information included tumor site, stage, treatment modality, time since diagnosis, and disease awareness. Current age at survey was categorized into four groups: 15–24, 25–29, 30–34, and ≥ 35 years. This classification was adapted from the 5-year age bands commonly used in adolescent and young adult cancer epidemiology, including 15–19, 20–24, 25–29, 30–34, and 35–39 years.^30^ In the present study, the 15–19 and 20–24 groups were combined because younger patients with gynecologic cancer were relatively few, consistent with the age-related epidemiological pattern of female-specific cancers.^31^ The final group was defined as ≥ 35 years rather than 35–39 years because AYA status was based on age at initial cancer diagnosis, whereas current age at survey could exceed 39 years for some participants. These age categories were used for descriptive purposes and for summarizing psychological outcome distributions, rather than for subgroup network comparison.

#### CS

CS were assessed using the Three-dimensional Inventory of Character Strengths (TICS), developed by Duan et al.^16^ This 15-item scale comprises three dimensions: Self-control, Inquisitiveness, and Caring (5 items per dimension). Items are rated on a five-point Likert scale ranging from 1 (very unlike me) to 5 (very like me). Dimension scores are calculated as the mean of the respective item scores, with higher scores indicating more prominent CS. The correspondence between TICS1–TICS15 and the 15 CS is shown in Table 1. The scale has demonstrated good psychometric properties, with a Cronbach's α coefficient exceeding 0.70 for the total scale and 0.79 for all sub-dimensions in previous studies.^16^ In this study, the TICS demonstrated good internal consistency, with a Cronbach's α coefficient of 0.847 for the total scale and 0.734, 0.809, and 0.854 for the Self-control, Inquisitiveness, and Caring subscales, respectively.

**Table 1:** Correspondence between TICS1–TICS15 and the 15 character strengths.

TICS code	CS	TICS code	CS
TICS1	Self-regulation	TICS9	Social intelligence
TICS2	Prudence	TICS10	Zest
TICS3	Judgment	TICS11	Fairness
TICS4	Perseverance	TICS12	Leadership
TICS5	Love of learning	TICS13	Teamwork
TICS6	Curiosity	TICS14	Honesty
TICS7	Humor	TICS15	Kindness
TICS8	Creativity	–	–

TICS, three-dimensional inventory of character strengths; CS, character strengths.

#### Psychological distress

Psychological distress was assessed using the Adolescent and Young Adult Psycho-Oncology Screening Tool (AYA-POST).^32^, ^33^, ^34^ This instrument comprises two components: a Distress Thermometer (DT) and a specific Problem List. The DT utilizes a visual analog scale ranging from 0 (“no distress”) to 10 (“extreme distress”) to assess participants’ distress over the past week. A score of 4 has been established as the optimal cutoff for identifying clinically significant distress in the Chinese version of the AYA-POST, with a sensitivity of 0.774 and an area under the curve of 0.736.^35^

#### Depression and anxiety

Anxiety and depression were evaluated using the Hospital Anxiety and Depression Scale (HADS).^36^ This 14-item instrument consists of two subscales: HADS-A for anxiety (7 items) and HADS-D for depression (7 items). Participants rate the frequency of symptoms over the past week on a four-point Likert scale ranging from 0 to 3, with higher scores indicating greater psychological outcome scores. Based on validation studies in cancer populations,^37^^,^^38^ the following cut-off scores were applied: a HADS-D score of ≥ 7 indicates a risk of depression (sensitivity: 0.86; specificity: 0.81),^37^ and a HADS-A score of ≥ 10 indicates a risk of anxiety.^38^ A total score of ≥ 15 suggests the presence of significant psychological distress. In this study, the HADS demonstrated excellent internal consistency, with a Cronbach's α coefficient of 0.927 for the total scale, 0.919 for the HADS-A subscale, and 0.835 for the HADS-D subscale.

### Statistical analysis

Descriptive statistics were computed using IBM SPSS version 27.0, while all network analyses were conducted within the R statistical environment (version 4.4.1). Descriptive statistics were used to summarize demographic and clinical characteristics. Categorical variables are presented as frequencies and percentages, and continuous variables as means and standard deviations. Univariate analyses were conducted to descriptively compare psychological distress, anxiety, and depression across demographic and clinical characteristics. For two-group comparisons, independent-samples *t* tests or Welch-corrected *t* tests were used for normally distributed data. For comparisons involving more than two groups, one-way analysis of variance was applied as appropriate. For treatment modalities, which were recorded as non-mutually exclusive variables, each category was compared between participants who did and did not receive that treatment. All tests were two-tailed, with statistical significance set at *P* < 0.05.

#### Gaussian graphical model and sensitivity analysis

To examine the multivariate conditional dependencies between CS and psychological outcomes (distress, anxiety, and depression), we estimated a GGM. In this network, nodes represent observed variables, and edges represent partial correlation coefficients after controlling for all other nodes in the network. To obtain a sparse and interpretable network structure, we applied the least absolute shrinkage and selection operator regularization method combined with the extended Bayesian information criterion. The tuning parameter was set to γ=0.5 to balance sensitivity and specificity, reduce potentially spurious connections, and retain the most prominent edges.^39^^,^^40^

For analyses requiring a community structure, two communities were defined a priori on theoretical grounds: a CS community (TICS1–TICS15) and a psychological-outcomes community (distress, anxiety, and depression). Node strength centrality, defined as the sum of the absolute edge weights connected to a node, was used as the primary indicator of structural prominence.^41^ Although betweenness and closeness centralities were calculated, they were interpreted cautiously and used only as supplementary descriptors. This decision aligns with recent methodological recommendations,^28^^,^^42^ which indicate that these metrics are often unstable in psychological networks and sensitive to variations in edge weights. The stability of the GGM was assessed using the bootnet package. Edge-weight accuracy was examined using 95% confidence intervals derived from nonparametric bootstrapping. The stability of centrality indices was assessed using the case-dropping bootstrap procedure and quantified using the correlation stability coefficient (CS-coefficient). A CS-coefficient of at least 0.25 was regarded as the minimum acceptable threshold for interpretation, with values above 0.50 indicating good stability.^28^ Additionally, bridge centrality was computed to characterize nodes contributing to connectivity between the CS and psychological outcomes communities.^43^

To examine the consistency of the primary GGM after accounting for demographic and clinical heterogeneity, a covariate-adjusted sensitivity analysis was conducted. Based on the univariate analyses and clinical relevance, financial dissatisfaction, time since diagnosis, family relationship satisfaction, and disease understanding were selected as covariates. Each network node was regressed on these covariates, and the standardized residuals were then used to re-estimate the GGM using the same EBICglasso procedure as in the primary analysis. The adjusted and primary networks were compared in terms of overall edge-weight similarity, node strength centrality, bridge strength centrality, and retention of key cross-community edges, particularly Curiosity–Depression and Zest–Distress.

#### Bayesian network analysis and sensitivity analysis

Given that the GGM is undirected, Bayesian network analysis was further conducted as a supplementary exploratory analysis to examine potential directed probabilistic dependencies among CS and psychological outcomes. Structure learning was performed using the hill-climbing algorithm and the Bayesian information criterion, with 1000 bootstrap replications,^44^ as implemented in the bnlearn R package.^45^ Directed edges were retained in the averaged network when their edge strength exceeded the optimal threshold determined using the method of Scutari et al.^46^ In line with the theoretical assumption that CS represent relatively stable positive psychological resources that may be associated with state-like psychological outcomes, the primary Bayesian network analysis applied a theory-informed blacklist. Specifically, directed edges from psychological-outcome nodes (psychological distress, anxiety, and depression) to the 15 CS nodes were prohibited before structure learning, whereas edges from CS to symptoms, within strengths, and within symptoms were permitted. This model was therefore interpreted as a theory-constrained exploratory Bayesian network rather than as an unconstrained test of causal direction. To assess the influence of this theoretical constraint, an additional unconstrained Bayesian network was estimated without the blacklist as a sensitivity analysis. The theory-constrained and unconstrained models were compared in terms of the number of retained edges, overlapping directed edges, and cross-module dependencies. Directed edges in both models were interpreted as probabilistic dependencies under the specified model assumptions rather than as evidence of temporal ordering or causal effects.

#### In silico simulation analysis

To explore the potential implications of the network findings under standardized hypothetical scenarios, we conducted an in silico simulation analysis. Network parameters were fitted using maximum likelihood estimation implemented in the bnlearn package. Before simulation, all variables were standardized to Z-scores to account for differences in measurement scales. For each CS node, we simulated a hypothetical 1-standard deviation (SD) increase by setting the node value from the model-implied baseline (Z = 0) to the hypothetical increased condition (Z = +1 SD). Posterior distributions of distress, anxiety, and depression were estimated using likelihood-weighted Monte Carlo sampling implemented with the bnlearn::cpdist function (method = “lw”), with 5000 samples generated for each simulation. Model-implied changes were calculated as the difference between the posterior mean under the hypothetical increased condition and the model-implied baseline condition. Overall model-implied change was calculated as the arithmetic mean of changes across distress, anxiety, and depression. To evaluate numerical stability, each simulation was repeated 100 times with 5000 Monte Carlo samples per run, and the standard deviation of the resulting model-implied changes was calculated as an indicator of Monte Carlo variability. These simulations were conducted for hypothesis generation only. Because they were based on cross-sectional data and a theory-constrained Bayesian network, they should not be interpreted as evidence of causal effects or real-world clinical intervention efficacy.^47^^,^^48^

#### Software implementation

GGM estimation and stability checks were performed using the bootnet and qgraph packages.^28^ Bridge centrality was calculated using networktools. Bayesian modeling and intervention simulations were performed using the bnlearn package.^45^ The package versions were bnlearn 5.1, bootnet 1.8, qgraph 1.9.8, and networktools 1.6.0. The bnlearn methodology was cited according to Scutari's original publication describing Bayesian network learning using the R package.

### Ethics approval and consent to participate

This study was approved by the Ethics Committee of the Third Xiangya Hospital, Central South University, Hunan Province, China (Approval No. 24766), and was conducted in accordance with the Declaration of Helsinki and its later amendments. Before participation, all eligible participants were informed of the study purpose, voluntary nature of participation, confidentiality of data, and their right to withdraw at any time without affecting their medical care. Adult participants provided informed consent before completing the questionnaire. For participants aged 15–17 years, informed consent was obtained from a parent or legal guardian, and assent was obtained from the adolescent participant.

## Results

### Characteristics

A total of 271 participants were included in the study. The majority of participants were married (68.63%) and had children (73.43%). In terms of clinical characteristics, most participants (62.73%) had been diagnosed within the past three months, and the most prevalent disease stage was Stage I (35.06%). Univariate analyses of psychological distress, anxiety, and depression across demographic and clinical characteristics are presented in Table 2. Anxiety scores differed significantly by financial dissatisfaction, insurance status, time since diagnosis, family relationship satisfaction, and disease understanding. Depression scores differed significantly by financial dissatisfaction, time since diagnosis, targeted/immunotherapy/hormone therapy, and family relationship satisfaction. In contrast, psychological distress scores did not differ significantly across the examined demographic and clinical characteristics. Descriptive statistics for CS and the prevalence of psychological distress, anxiety, and depression are provided in Supplementary Tables S1 and S2, respectively.

**Table 2:** Univariate analyses of demographic and clinical characteristics associated with psychological distress, anxiety, and depression (*N* = 271).

Characteristics	*n* (%)	DT [M (SD)]	*t/F*	*P* value	Anxiety [M (SD)]	*t/F*	*P* value	Depression [M (SD)]	*t/F*	*P* value
Age (mean ± SD, years)	34.75 ± 5.78									
Age group (years)			0.073	0.975		0.297	0.828		0.581	0.628
15–24	20 (7.38)	3.90 (2.49)			7.05 (5.58)			5.30 (3.85)		
25–29	22 (8.12)	4.18 (2.84)			7.00 (4.93)			6.41 (5.79)		
30–34	59 (21.77)	4.08 (2.88)			7.27 (4.51)			4.95 (3.99)		
≥ 35	170 (62.73)	3.96 (2.60)			6.59 (5.13)			5.48 (4.57)		
Marital status			0.498	0.609		0.41	0.664		0.309	0.735
Single	51 (18.82)	4.18 (2.78)			7.33 (5.20)			5.73 (4.77)		
Married	186 (68.63)	3.90 (2.58)			6.63 (5.08)			5.44 (4.58)		
Divorced/Widowed	34 (12.55)	4.32 (2.96)			6.97 (4.28)			4.94 (3.69)		
Fertility status			−0.899	0.371		−1.499	0.137		−0.465	0.642
Parous	199 (73.43)	3.91 (2.64)			6.53 (4.91)			5.35 (4.52)		
Nulliparous	72 (26.57)	4.25 (2.74)			7.58 (5.20)			5.64 (4.47)		
Education			0.121	0.947		0.525	0.665		0.914	0.434
Junior high school or below	82 (30.26)	3.88 (2.68)			6.57 (4.92)			5.22 (4.27)		
Senior high school or vocational secondary school	81 (29.89)	4.09 (2.86)			7.38 (5.58)			5.85 (5.02)		
Junior college diploma	50 (18.45)	4.12 (2.58)			6.68 (4.48)			5.88 (4.44)		
Undergraduate or above	58 (21.40)	3.97 (2.48)			6.45 (4.73)			4.74 (4.10)		
Employment status			−0.752	0.453		−1.519	0.13		−1.505	0.134
Employed	118 (43.54)	3.86 (2.72)			6.28 (5.17)			4.96 (4.61)		
Unemployed	153 (56.46)	4.11 (2.62)			7.22 (4.85)			5.79 (4.40)		
Dissatisfied with current financial status			1.304	0.269		7.75	< 0.001		6.161	< 0.001
Strongly disagree	32 (11.81)	3.88 (2.78)			5.00 (4.30)			5.28 (4.41)		
Disagree	59 (21.77)	3.41 (2.51)			4.46 (3.89)			3.34 (3.46)		
Neutral	108 (39.85)	4.19 (2.86)			8.25 (4.90)			6.65 (4.65)		
Agree	52 (19.19)	4.46 (2.30)			7.75 (5.42)			5.87 (4.72)		
Strongly agree	20 (7.38)	3.80 (2.57)			6.40 (5.30)			4.10 (3.71)		
Insurance status			1.394	0.169		−3	0.004		−1.162	0.251
No	36 (13.28)	4.47 (2.06)			4.72 (4.39)			4.67 (4.17)		
Yes	235 (86.72)	3.93 (2.74)			7.13 (5.02)			5.54 (4.55)		
Residence			0.85	0.397		−1.09	0.277		−1.61	0.109
Urban	170 (62.73)	4.11 (2.59)			6.56 (5.16)			5.09 (4.58)		
Rural	101 (37.27)	3.82 (2.79)			7.23 (4.72)			5.99 (4.34)		
Time since diagnosis			2.219	0.067		2.738	0.029		3.647	0.007
< 1 month	81 (29.89)	4.28 (2.66)			7.47 (5.45)			5.79 (4.86)		
1–3 months	89 (32.84)	3.81 (2.48)			6.29 (4.33)			5.04 (3.83)		
3 months–1 year	50 (18.45)	3.24 (2.56)			5.40 (4.62)			3.86 (3.70)		
1–3 years	26 (9.59)	4.46 (3.08)			7.27 (5.42)			6.85 (4.88)		
> 3 years	25 (9.23)	4.84 (2.79)			8.84 (5.35)			7.28 (5.57)		
Disease stage			0.664	0.618		0.649	0.628		0.728	0.574
I	95 (35.06)	3.79 (2.67)			6.36 (4.72)			4.88 (4.13)		
II	44 (16.24)	3.84 (2.68)			6.45 (4.42)			5.25 (4.35)		
III	42 (15.50)	3.90 (2.45)			7.69 (5.56)			6.10 (4.30)		
IV	28 (10.33)	4.14 (2.82)			6.79 (6.14)			5.82 (5.68)		
Uncertain	62 (22.88)	4.45 (2.74)			7.16 (4.90)			5.76 (4.73)		
Therapeutic method										
Surgery			0.355	0.724		−0.121	0.904		0.911	0.365
No	57 (21.03)	4.12 (2.92)			6.74 (5.02)			5.93 (4.74)		
Yes	214 (78.97)	3.97 (2.60)			6.83 (5.01)			5.29 (4.44)		
Chemotherapy			1.3	0.195		1.868	0.063		1.042	0.298
No	117 (43.17)	4.25 (2.80)			7.46 (5.16)			5.76 (4.82)		
Yes	154 (56.83)	3.82 (2.55)			6.31 (4.84)			5.18 (4.25)		
Targeted / Immunotherapy / Hormone therapy			−1.065	0.294		−1.611	0.116		−2.704	0.01
No	241 (88.93)	3.94 (2.66)			6.63 (4.94)			5.16 (4.41)		
Yes	30 (11.07)	4.50 (2.71)			8.27 (5.30)			7.60 (4.69)		
Others			−0.4	0.696		−1.462	0.169		−0.644	0.532
No	259 (95.57)	3.99 (2.66)			6.72 (5.00)			5.38 (4.46)		
Yes	12 (4.43)	4.33 (2.93)			8.75 (4.69)			6.42 (5.48)		
Cancer type			1.02	0.384		1.032	0.379		0.108	0.956
Cervical	122 (45.02)	4.11 (2.78)			7.08 (5.14)			5.47 (4.34)		
Endometrial	36 (13.28)	3.69 (2.56)			6.28 (5.43)			5.19 (5.07)		
Ovarian	105 (38.75)	3.88 (2.57)			6.88 (4.84)			5.51 (4.67)		
Other	8 (2.95)	5.38 (2.33)			4.12 (1.13)			4.75 (1.75)		
Family relationship satisfaction			1.885	0.132		3.93	0.009		3.579	0.014
Dissatisfied	16 (5.90)	4.88 (2.25)			9.44 (4.87)			7.94 (4.86)		
Neutral	29 (10.70)	4.83 (2.42)			8.31 (4.42)			6.66 (4.41)		
Satisfied	112 (41.33)	3.92 (2.54)			7.02 (5.14)			5.53 (4.62)		
Very satisfied	114 (42.07)	3.75 (2.85)			5.85 (4.82)			4.67 (4.20)		
Disease understanding			2.202	0.088		5.408	0.001		2.517	0.059
Unfamiliar	29 (10.70)	4.62 (3.14)			6.55 (6.23)			6.03 (6.14)		
Neutral	52 (19.19)	4.58 (2.77)			8.77 (5.35)			6.42 (4.80)		
Familiar	144 (53.14)	3.85 (2.46)			6.79 (4.58)			5.39 (4.18)		
Very familiar	46 (16.97)	3.43 (2.73)			4.80 (4.28)			4.04 (3.63)		
Family history of cancer			−0.417	0.679		−0.361	0.72		0.602	0.551
Yes	32 (11.81)	3.81 (2.78)			6.47 (5.76)			5.94 (5.19)		
No	239 (88.19)	4.03 (2.65)			6.85 (4.90)			5.36 (4.41)		

DT, Distress Thermometer; SD, standard deviation. Independent-samples *t* tests, or one-way analysis of variance were used as appropriate. For treatment modalities, each category was compared between patients who did and did not receive that treatment because treatment modalities were not mutually exclusive. A positive *t*-value indicates that the first group had a higher mean score than the second group, whereas a negative *t*-value indicates the opposite.

### Gaussian graphical model estimation and sensitivity analysis

#### Network and centrality

The overall network structure, estimated using the GGM, is shown in Fig. 1. The analysis examined two prespecified communities: the CS community and the psychological-outcome community. The CS indicators (TICS1–TICS15; see Table 1 for the corresponding CS) clustered into a highly interconnected subsystem characterized by numerous moderate-to-strong partial correlations. Similarly, the psychological-outcome nodes, including distress, anxiety, and depression, formed a closely connected symptom module, within which the edge between anxiety and depression was the strongest. In contrast to the dense within-community connections, cross-community edges were relatively sparse and primarily involved Zest (TICS10) and Curiosity (TICS6).

**Fig. 1:**
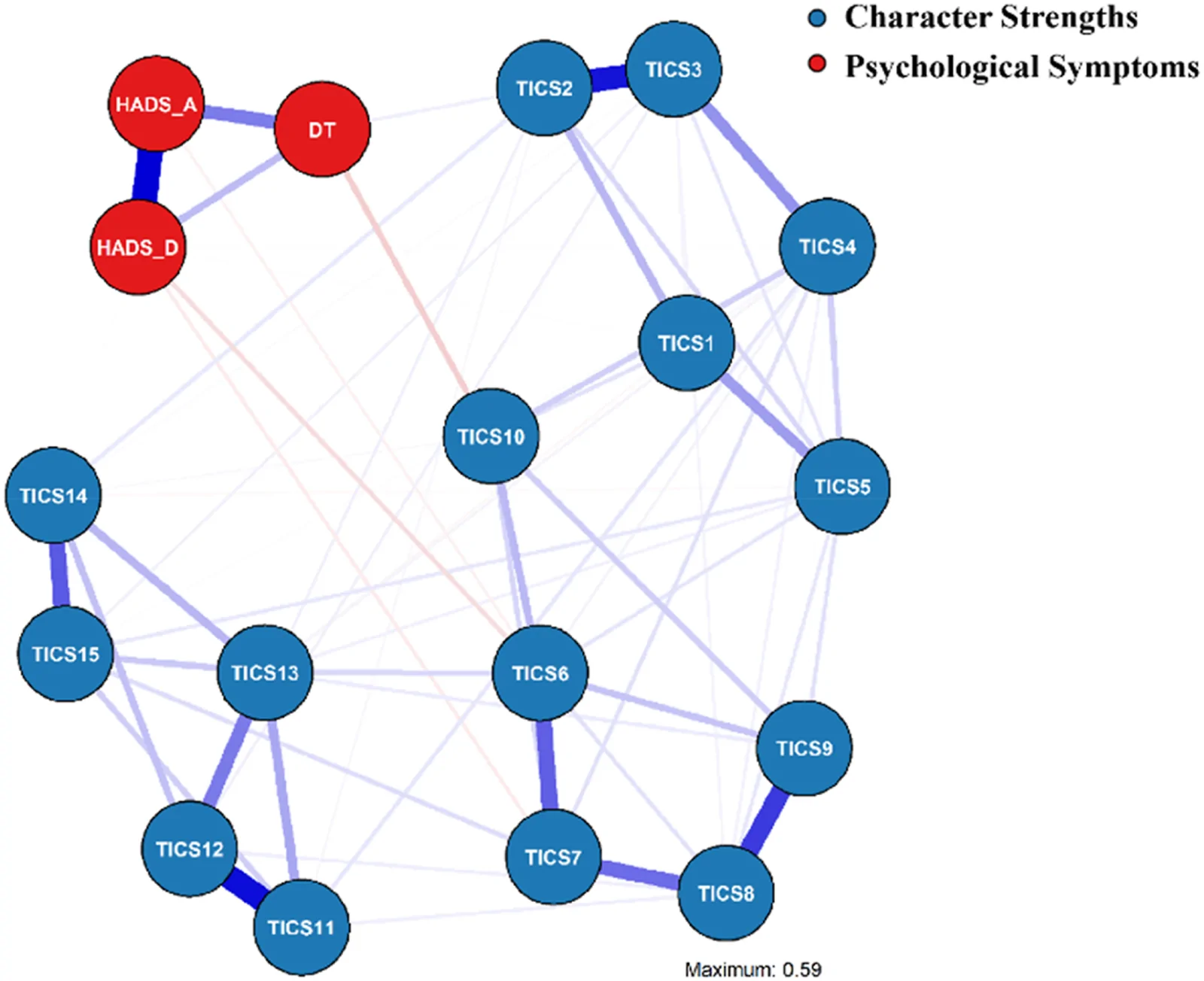
Gaussian graphical model of character strengths and psychological outcomes. Edges represent regularized partial correlations; blue edges indicate positive associations and red edges indicate negative associations. Edge thickness and color saturation reflect the strength of the association. Communities were defined by construct domain, with TICS nodes comprising the character strengths community and DT/HADS-A/HADS-D comprising the psychological symptoms community. TICS, three-dimensional inventory of character strengths; DT, Distress Thermometer; HADS-A, Hospital Anxiety and Depression Scale for anxiety; HADS-D, Hospital Anxiety and Depression Scale for depression.

#### Node centrality

The CS nodes generally had higher strength centrality values within the estimated network (Fig. 2). Leadership (TICS12, strength = 1.119) had the highest node strength, followed by Creativity (TICS8, 1.052), Curiosity (TICS6, 1.016), and Teamwork (TICS13, 1.007). The psychological-outcome nodes had moderate or lower strength centrality values: anxiety (HADS-A = 0.925), depression (HADS-D = 0.881), and psychological distress (DT = 0.604). Betweenness and closeness values are presented in Fig. 2 for descriptive completeness only. The case-dropping bootstrap shown in Supplementary Fig. S1 yielded a correlation stability coefficient of 0.517 for node strength, meeting the prespecified interpretability threshold. Closeness (CS-coefficient = 0.207) and betweenness (CS-coefficient = 0.000) were below 0.25. Subsequent interpretation focused on node strength and bridge strength as structural descriptors of the estimated network.

**Fig. 2:**
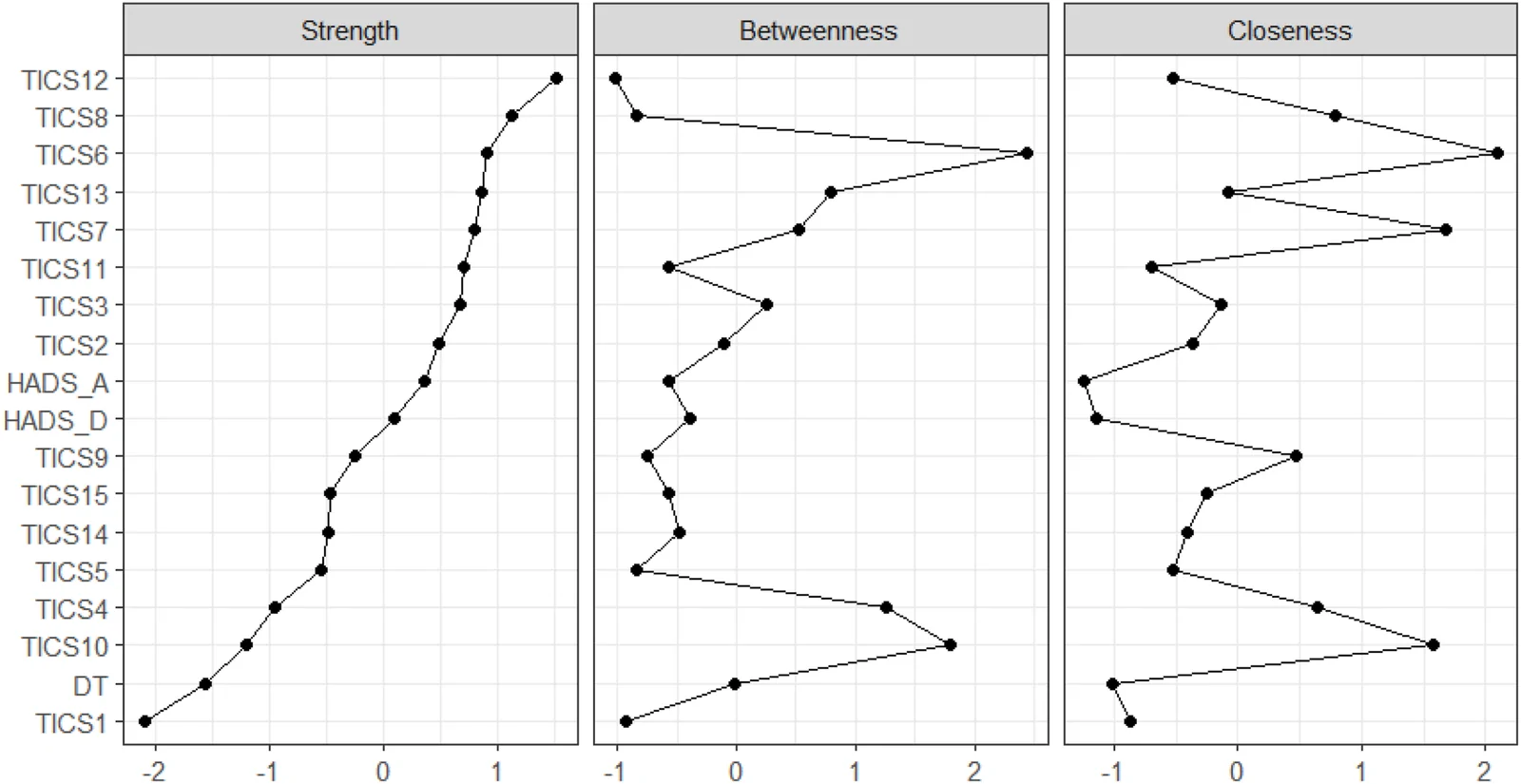
Node centrality indices in the combined network of distress, anxiety, depression, and TICS item-level character strengths. TICS, three-dimensional inventory of character strengths; DT, Distress Thermometer; HADS-A, Hospital Anxiety and Depression Scale for anxiety; HADS-D, Hospital Anxiety and Depression Scale for depression. Strength, betweenness, and closeness centrality are shown for all nodes. Higher values indicate greater relative centrality within the estimated network. Centrality indices are displayed as standardized z scores.

#### Bridge centrality

Bridge centrality analysis described cross-community connectivity between the two prespecified construct domains—the CS community and the psychological-outcomes community (Supplementary Fig. S2). Psychological distress had the highest bridge strength value among the psychological-outcome nodes (0.148), followed by depression (0.136) and anxiety (0.034). Among CS, Curiosity (TICS6) and Zest (TICS10) had the highest bridge strength values (both = 0.112), while Humor (TICS7, 0.046) showed a smaller cross-community value. Bridge strength values for the remaining nodes approached zero, indicating limited cross-community connectivity within this estimated network.

#### Covariate-adjusted sensitivity analysis

The covariate-adjusted sensitivity analysis showed high similarity between the primary GGM and the model estimated after adjustment for financial dissatisfaction, time since diagnosis, family relationship satisfaction, and disease understanding. Pearson and Spearman correlations of edge weights between the two networks were 0.995 and 0.942, respectively. Strength centrality and bridge strength rankings were also similar, with Spearman correlations of 0.973 and 0.989, respectively. The Curiosity–Depression and Zest–Distress cross-community edges were retained after adjustment and remained negative. The Curiosity–Depression edge changed from −0.078 to −0.065, and the Zest–Distress edge changed from −0.112 to −0.109 (Supplementary Table S3). These findings indicate consistency under this specific covariate-adjustment procedure.

### Bayesian network analysis

#### Theory-constrained model

In the theory-constrained Bayesian network, the optimal edge strength threshold was 0.500. Directed edges appearing in more than 50.0% of the 1000 bootstrap samples were therefore retained. The bootstrap-averaged network contained 31 directed edges under the predefined theoretical constraint (Fig. 3).

**Fig. 3:**
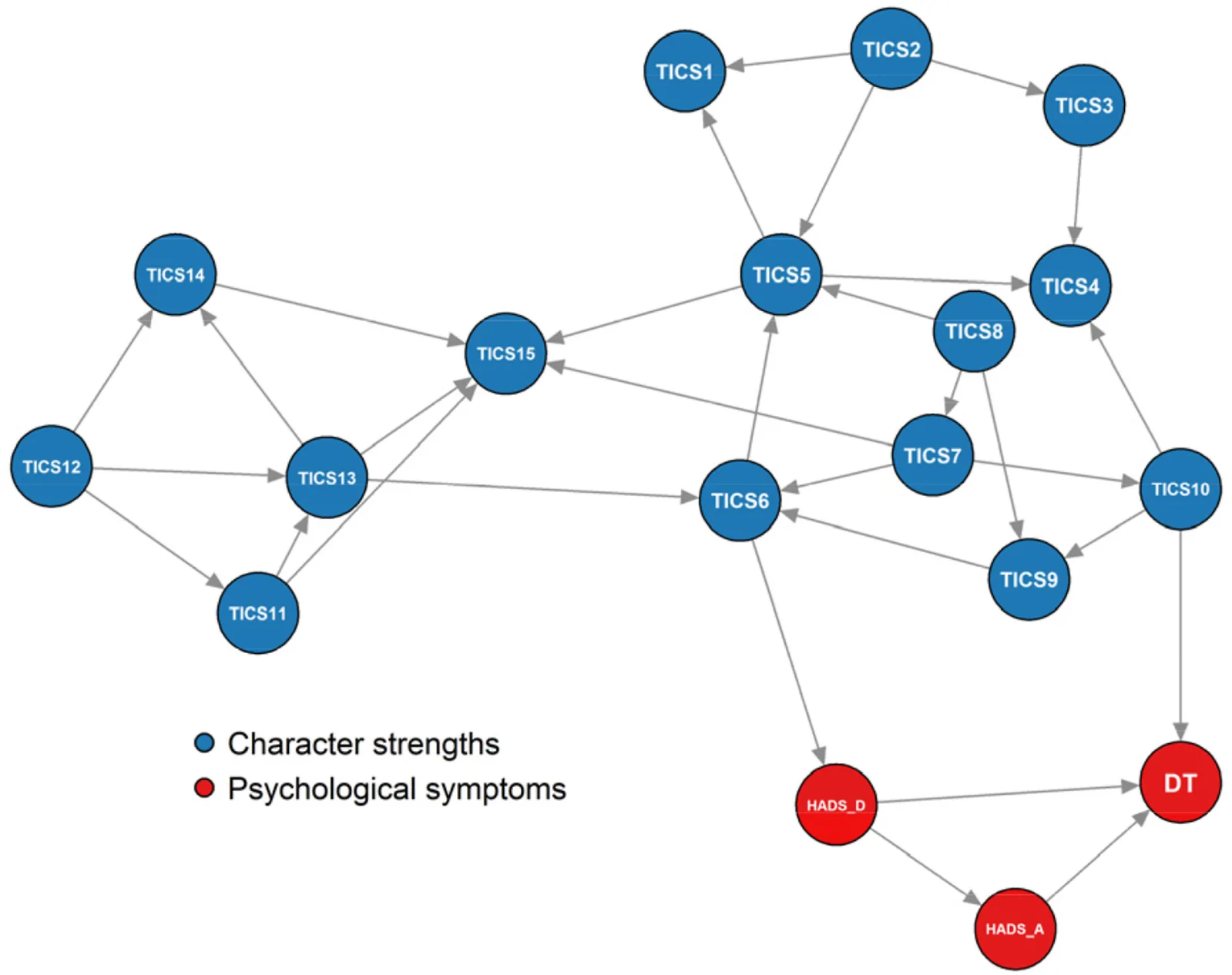
Theory-constrained Bayesian network model for character strengths, psychological distress, anxiety, and depression. TICS, three-dimensional inventory of character strengths; DT, Distress Thermometer; HADS-A, Hospital Anxiety and Depression Scale for anxiety; HADS-D, Hospital Anxiety and Depression Scale for depression. Arrows denote estimated probabilistic dependencies in the fitted Bayesian network, oriented from parent to child nodes as defined within the network structure; they do not represent causal effects.

Most retained edges were located within the CS community, indicating dense dependency patterns among CS indicators. Within the psychological-outcomes community, three directed dependencies were retained: depression to anxiety (HADS-D→HADS-A; strength = 1.000, direction = 0.579), anxiety to distress (HADS-A→DT; strength = 1.000, direction = 0.662), and depression to distress (HADS-D→DT; strength = 0.576, direction = 0.745).

Cross-module dependencies were sparse. Two directed edges from CS to psychological outcomes were retained in the theory-constrained model (Supplementary Table S4): Zest to distress (TICS10→DT; strength = 0.645, direction = 1.000) and Curiosity to depression (TICS6→HADS-D; strength = 0.572, direction = 1.000).

The node-level degree results (Supplementary Table S5) showed that TICS2, TICS8, and TICS12 had an in-degree of 0 and an out-degree of 3, while TICS7, TICS10, TICS11, and HADS-D had relatively high out-degree values. These degree patterns indicated greater outgoing connectivity for these nodes within the learned theory-constrained graph.

#### Unconstrained model

In the unconstrained Bayesian network (Supplementary Fig. S3), the optimal edge strength threshold was 0.516; retained edges therefore appeared in at least 51.6% of the bootstrap samples. The averaged network contained 31 directed edges. Compared with the theory-constrained model, 28 directed edges overlapped between the two models, indicating substantial similarity in overall edge composition. The unconstrained model also showed sparse cross-module dependencies, with two cross-module edges retained (Supplementary Table S6). The Curiosity–depression dependency was retained with the same orientation as in the theory-constrained model (TICS6→HADS-D; strength = 0.624, direction = 0.672), although the estimated direction probability was moderate. By contrast, the orientation of the Zest–distress dependency was sensitive to the blacklist specification. Whereas the theory-constrained model retained the edge from Zest to distress (TICS10→DT), the unconstrained model retained the edge from distress to Zest (DT→TICS10; strength = 0.738, direction = 0.633). Taken together, the two models showed substantial overlap in overall edge composition, while the orientation of the Zest–distress dependency remained sensitive to model specification.

### In silico intervention

The results of the in silico simulation analysis are presented in Fig. 4 and Supplementary Table S7. Curiosity (TICS6) was the CS node associated with the largest model-implied outcome change. Specifically, a hypothetical 1-SD increase in Curiosity corresponded to the largest model-implied change toward lower overall psychological-outcome scores (−0.230 SD). A hypothetical 1-SD increase in Curiosity was associated with model-implied changes of −0.306 SD for depression, −0.218 SD for anxiety, and −0.167 SD for distress. Hypothetical increases in Humor (TICS7) and Creativity (TICS8) corresponded to smaller model-implied changes in overall psychological outcome scores (−0.110 SD and −0.078 SD, respectively). Regarding outcome-specific patterns, the model-implied change for Zest (TICS10) was primarily observed for psychological distress (−0.123 SD), whereas the corresponding model-implied changes for anxiety and depression were close to zero (0.013 SD and 0.016 SD, respectively). Monte Carlo simulations demonstrated high numerical stability, with standard deviations of overall model-implied changes ranging from 0.015 to 0.019 SD across CS nodes.

**Fig. 4:**
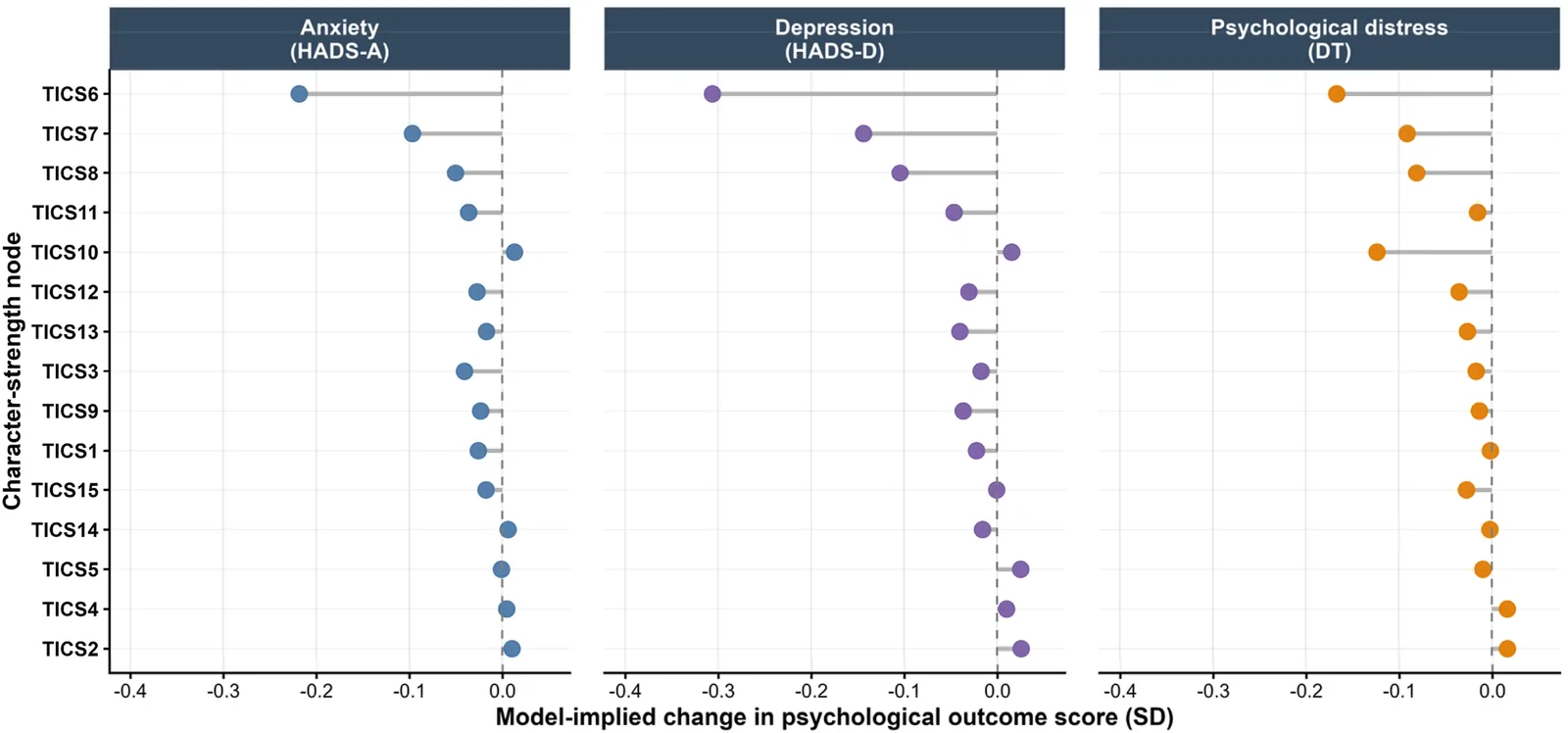
Computational simulation results for hypothetical 1-SD increases in character strength nodes. TICS, three-dimensional inventory of character strengths; DT, Distress Thermometer; HADS-A, Hospital Anxiety and Depression Scale for anxiety; HADS-D, Hospital Anxiety and Depression Scale for depression; SD, standard deviation. Negative values indicate lower estimated symptom levels. Results represent model-implied changes under specified assumptions and do not establish causal or clinical intervention effects.

## Discussion

### Main findings

The present study showed that CS were associated with psychological distress, anxiety, and depression in a specific rather than generalized manner among AYGC. The GGM indicated that the two prespecified CS and psychological outcomes communities showed dense within-community and sparse between-community connections, with Curiosity and Zest showing the highest bridge strengths among the CS nodes and psychological distress showing the highest bridge strength among the psychological-outcome nodes. Bayesian network analysis further suggested sparse cross-module probabilistic dependencies: the Curiosity–depression dependency was retained across both theory-constrained and unconstrained models, whereas the orientation of the Zest–distress dependency was sensitive to model specification. In silico intervention provided additional hypothesis-generating evidence that Curiosity showed broader model-implied outcome changes, while Zest showed a more distress-specific model-implied pattern. Taken together, these findings provide a preliminary basis for further investigation of strength–symptom specificity and symptom-relevant directions for psychosocial research in AYGC.

The GGM showed a network structure in which CS and psychological outcomes formed two highly clustered yet relatively distinct modules connected through sparse, specific nodes. Psychological distress had the highest bridge centrality among the psychological-outcome nodes, indicating greater cross-community connectivity within the estimated network rather than a causal or downstream role. This finding aligns with prior systematic reviews showing that psychological distress frequently coexists with, and is closely associated with, anxiety and depressive symptoms in AYGC.^49^ That literature, however, reported prevalence and associations rather than conditional associations among construct-level psychological outcomes, which the present network framework addresses. Consistent with the NCCN definition, distress in this context is not merely a symptom but a multidimensional construct involving physical, emotional, and spiritual components.^50^ It has also been associated with functional impairment and more severe psychopathology,^51^ which may help explain its prominent cross-community position in the estimated network.

Node-specific analyses indicated that associations between CS and psychological outcomes were specific rather than generalized. Curiosity (TICS6) and Zest (TICS10) had the highest bridge strength values among the CS nodes, indicating relatively greater cross-community connectivity within the estimated network. This finding parallels network analyses in nonclinical and other health-compromised populations, where Zest has also shown a prominent network position and associations with positive health behaviors and lower negative affect.^24^^,^^52^ Whereas Zest may represent an energetic correlate, Curiosity may represent a more cognitively oriented one. Although direct research on Curiosity in cancer networks is limited, behavioral studies suggest that information seeking and active learning—hallmarks of Curiosity—may be relevant to coping with cancer-related cognitive impairment and uncertainty.^53^^,^^54^ To the extent that Curiosity is associated with active cognitive engagement rather than passive emotional regulation, it may be related to greater acceptance of uncertainty and lower perceived burden,^55^ which could represent an adaptive correlate in the unpredictable context of cancer.

Bayesian network analysis provided an exploratory, model-based representation of potential directed probabilistic dependencies rather than definitive evidence of causal direction. In the theory-constrained model, Zest and Curiosity were connected with psychological-outcome nodes under predefined assumptions. However, sensitivity analysis showed different levels of consistency across model specifications: the Curiosity–depression dependency was retained across models, whereas the orientation of the Zest–distress dependency changed in the unconstrained model. These findings are broadly compatible with, but do not confirm, vulnerability-oriented perspectives suggesting that positive traits may be associated with lower psychological outcomes.^56^ This is also consistent with network studies based on the Big Five personality traits, which suggest that positive traits such as Conscientiousness and Extraversion may be associated with specific symptoms.^57^ Longitudinal evidence also suggests that strengths of moral character are prospectively associated with lower odds of depression.^25^ Nevertheless, because the present study was cross-sectional and partly theory-constrained, the Bayesian network findings should be interpreted as hypothesis-generating rather than as confirmation that participants’ core personality resources temporally or causally precede symptom improvement.

Accordingly, the present findings should not be interpreted as evidence for the absence of a “scar effect.” In the theory-constrained model, directed edges from psychological outcomes to CS were prohibited by design, and the unconstrained model showed that the Zest–distress dependency could also be oriented from distress to Zest. Therefore, possible reverse or reciprocal relationships between psychological outcomes and CS cannot be ruled out. Research indicates that substantial personality changes may be associated with long-term, cumulative stress,^26^ whereas in earlier phases of illness, positive psychological resources may remain relatively available and may be associated with better adjustment.^58^ Furthermore, specific strengths such as Curiosity, which is conceptually related to Openness, may reflect cognitive engagement, information seeking, and openness to uncertainty, which could be relevant to adaptation in the cancer context.^26^ Additionally, Zest may be closely connected with vitality, engagement, and perceived energy during treatment; Conservation of Resources theory^59^ suggests that threats to valued resources may be associated with efforts to maintain daily functioning and meaning, as described qualitatively in young people with cancer.^60^ However, given that the orientation of the Zest–distress dependency differed between the two Bayesian network specifications, this relationship should be interpreted as a conditional cross–module association rather than as a confirmed unidirectional protective pathway.^20^

Finally, the in silico simulation analysis extended the static network findings by generating preliminary, model-implied hypotheses regarding the symptom-related patterns of the identified bridge strengths. Under the assumptions of the estimated model, a hypothetical increase in Curiosity corresponded to model-implied changes toward lower levels of distress, anxiety, and depression, suggesting a relatively broad model-implied psychological-outcome pattern. This pattern may be compatible with broaden-and-build processes involving cognitive exploration and meaning making, which have been applied clinically in other cancer populations^61^ although such processes were not directly examined in the present study. Intolerance of uncertainty may also warrant investigation as a potential explanatory factor linking Curiosity with anxiety and depression.^62^ In contrast, Zest showed a more outcome-specific pattern, with the model-implied change primarily observed for psychological distress. This pattern may be relevant to vitality, engagement, cancer-related fatigue, and somatic burden in AYGC,^63^ but the present analysis cannot determine the underlying processes or establish that Zest directly influences these outcomes. Conversely, social strengths such as Leadership and Teamwork showed smaller model-implied changes in simulations, possibly reflecting social isolation and role disruption,^64^ which may restrict the functional utility of these traits in the acute clinical context.^65^

### Implications for nursing practice and research

The findings should broaden, rather than replace, routine oncology nursing assessment. Within established psychosocial assessment, nurses may use brief strength-sensitive questions alongside routine assessment of distress, treatment-related symptoms, information needs, functioning, and unmet supportive-care needs.^32^, ^33^, ^34^, ^35^^,^^50^ Questions about what patients still wish to understand, which activities or roles remain meaningful, and what has previously helped them cope may help identify existing strengths and their potential use in the current clinical context. In patient-centered communication and supportive care planning, the findings may inform how nurses help patients recognize and use existing strengths rather than which strength should be increased. For Curiosity, nurses may support manageable information seeking and shared decision-making according to patients' preferences and readiness. For Zest, nurses may explore which activities, relationships, or roles continue to provide energy or meaning and support gradual re-engagement when clinically appropriate. Reduced vitality or participation should first prompt assessment of treatment toxicity, cancer-related fatigue, pain, sleep disturbance, depression, and practical barriers.^63^ Nurses may then co-design low-burden actions that fit the patient's symptom burden, treatment phase, functional capacity, and priorities. Clinically significant distress should continue to trigger established supportive-care and referral pathways to psycho-oncology or other qualified professionals.^50^

For research, Curiosity and Zest should be treated as candidate areas for future nurse-led or nurse-supported intervention development rather than established intervention components. Longitudinal and intensive within-person studies are needed to clarify temporal ordering and contextual variation. Co-designed feasibility studies could then test preference-tailored information support for Curiosity and graded re-engagement in meaningful activities for Zest, followed by controlled trials evaluating clinical outcomes, potential mediators, implementation burden, and unintended pressure to appear positive.

### Strengths and limitations

Several features strengthen this study. First, the focus on AYGC addresses an understudied population whose psychological burden intersects with cancer treatment and key developmental concerns. Second, it integrates the GGM, Bayesian network analysis, and in silico intervention analysis, enabling complementary examination of conditional associations, assumption-dependent probabilistic dependencies, and model-implied hypotheses regarding candidate strength-related nodes. Third, by characterizing Curiosity and Zest as strength-related nodes with distinct symptom-related patterns, the study provides a more nuanced basis for future strength-based psychosocial research than analyses using only aggregate strength or distress scores.

Several limitations necessitate caution in interpreting these results. First, the cross-sectional design precludes causal inference. Although Bayesian network analysis and in silico simulation analysis provided exploratory information on directed probabilistic dependencies and hypothetical model-implied changes, these findings were dependent on the specified model assumptions and do not establish temporal ordering, causal effects, or clinical intervention efficacy. Reverse or reciprocal relationships between CS and psychological outcomes cannot be excluded. Second, participants were recruited from only two tertiary hospitals in Hunan Province, China, which may limit the generalizability of the findings to other regions, health care settings, and sociocultural contexts. Third, the age distribution was uneven, with relatively few younger participants and a larger proportion aged ≥ 35 years. This may reflect the age-related distribution of gynecologic cancers within the AYA population. Therefore, age-stratified analyses and network comparisons across age subgroups were not feasible. Fourth, all variables were assessed using self-report questionnaires and may be subject to recall and social desirability biases. Selection bias also cannot be excluded, as patients with greater psychological vulnerability—or, conversely, stronger resilience—may have been less likely to participate, and no objective physiological markers were available to corroborate the self-reported data. Fifth, the estimated networks represented group–level associations that, owing to non-ergodicity, may not reflect within-person symptom dynamics. Unmeasured demographic, clinical, or psychosocial factors may also have influenced the observed associations. Finally, the candidate strength-related nodes and model-implied outcome changes were derived from statistical simulations rather than direct clinical intervention data. Whether changes in Curiosity or Zest temporally precede or contribute to changes in distress, anxiety, or depression requires confirmation in longitudinal and controlled intervention studies.

## Conclusions

In conclusion, CS and psychological outcomes formed relatively distinct but sparsely connected communities in AYGC. Curiosity and Zest emerged as candidate strength-related nodes with distinct model-dependent patterns. The Curiosity–depression dependency was retained across both Bayesian network specifications, whereas the orientation of the Zest–distress dependency differed by model specification. In silico simulation further indicated broader model-implied psychological-outcome changes for Curiosity and a more distress-specific pattern for Zest. These hypothesis-generating findings support further longitudinal and intervention research before their temporal ordering, clinical relevance, or utility for intervention development can be determined.

## CRediT authorship contribution statement

**Ning Qin** and **Yuxuan Li**: Conceptualization, Investigation, Data curation, Formal analysis, Writing–Original draft, Writing–review & editing. **Juan Luo** and **Dongren Qin**: Investigation, Data curation. **Jie Zhu** and **Huiyi Zhang**: Methodology, Writing-review & editing. **Jian Peng and Zitong Lu**: Validation, Methodology, Writing–review & editing. **Jianfei Xie** and **Shuijing Yi**: Conceptualization, Methodology, Resources, Supervision, Funding acquisition, Project administration, Writing–review and Editing. **Peter H. F. Ng**: Supervision, Resources. All authors have read and approved the final manuscript.

## Ethics statement

This study was approved by the Ethics Committee of the Third Xiangya Hospital, Central South University, Hunan Province, China (Approval No. 24766) and was conducted in accordance with the 1964 Helsinki Declaration and its later amendments or comparable ethical standards. All participants provided informed consent. For participants aged 15–17 years, informed consent was obtained from a parent or legal guardian, and assent was obtained from the adolescent participant.

## Data availability

The data are available from the corresponding author upon reasonable request but are not publicly available because they contain information that could compromise participant privacy.

## Declaration of generative AI and AI-assisted technologies in the writing process

During the preparation of this work, the authors used ChatGPT (OpenAI) to improve the language, grammar, and readability of the manuscript. After using this tool, the authors reviewed and edited the content as needed and take full responsibility for the content of the publication.

## Funding

This study was supported by the Major Special Project of Health Science Research in Hunan Province (Grant No. 20256491) and the Fundamental Research Funds for the Central Universities of Central South University (Grant No. 202622TS0657). The funder had no role in the study design; collection, analysis, or interpretation of data; writing of the report; or the decision to submit the article for publication.

## Declaration of competing interest

The authors declare no conflict of interest. The corresponding author, Prof. Jianfei Xie, is an editorial board member of *Asia–Pacific Journal of Oncology Nursing*. The article was subject to the journal's standard procedures, with peer review handled independently of Prof. Xie and the research groups.
