# Supplementary material for: Character strengths, psychological distress, anxiety, and depression in adolescents and young adults with gynecologic cancer: A network analysis
Source: Asia Pac J Oncol Nurs. 2026 Sep 10;13:101027. doi: 10.1016/j.apjon.2026.101027 (PMC13634094; doi:10.1016/j.apjon.2026.101027)
Supplement: Supplementary file 1 [file mmc1.docx]

**Supplementary Figs. S1-3.**

**Content**

Fig. S1. Centrality stability analysis of the psychological symptom and CS network

Fig. S2. Bridge centrality metrics for the network of character strengths, psychological distress, anxiety, and depression

Fig. S3. Unconstrained Bayesian network model for character strengths, psychological distress, anxiety, and depression

**Fig. S1.** Centrality stability analysis of the psychological symptom and CS network

**Note.** Correlations of centrality indices across varying proportions of sampled cases; Green dashed line: CS = 0.5; Blue dashed line: CS = 0.7.

**Fig. S2.** Bridge centrality metrics for the network of character strengths, psychological distress, anxiety, and depression

**Fig. S3.** Unconstrained Bayesian network model for character strengths, psychological distress, anxiety, and depression

**Note.** Directed arrows indicate the estimated direction of probabilistic dependence between connected nodes, with the arrow pointing from the putative parent node to the child node.
