# Supplementary material for: Character strengths, psychological distress, anxiety, and depression in adolescents and young adults with gynecologic cancer: A network analysis
Source: Asia Pac J Oncol Nurs. 2026 Sep 10;13:101027. doi: 10.1016/j.apjon.2026.101027 (PMC13634094; doi:10.1016/j.apjon.2026.101027)
Supplement: Supplementary file 2 [file mmc3.docx]

**Supplementary Tables. S1-7.**

**Content**

Table S1. Descriptive statistics of TICS item-level character strengths in AYGC

Table S2. Prevalence of psychological distress, anxiety, and depression by demographic and clinical characteristics（n = 271）

Table S3. Covariate-adjusted sensitivity analysis of the Gaussian graphical model

Table S4. Stable directed edges in the theory-constrained Bayesian network

Table S5. In-degree and out-degree characteristics of all nodes in the theory-constrained Bayesian network

Table S6. Stable directed edges in the unconstrained Bayesian network

Table S7. Model-implied changes in psychological outcomes following hypothetical 1-SD increases in character-strength nodes

**Table S1.** Descriptive statistics of TICS item-level character strengths in AYGC

| Variable | Range | Min | Max | Mean item score  (Mean ± SD) |
| --- | --- | --- | --- | --- |
| **TICS items** |  |  |  |  |
| TICS1 | 1–5 | 1 | 5 | 2.86 ± 1.00 |
| TICS2 | 1–5 | 1 | 5 | 3.26 ± 0.97 |
| TICS3 | 1–5 | 1 | 5 | 3.28 ± 1.02 |
| TICS4 | 1–5 | 1 | 5 | 3.52 ± 0.95 |
| TICS5 | 1–5 | 1 | 5 | 3.04 ± 1.09 |
| TICS6 | 1–5 | 1 | 5 | 3.48 ± 0.98 |
| TICS7 | 1–5 | 1 | 5 | 3.34 ± 0.98 |
| TICS8 | 1–5 | 1 | 5 | 3.36 ± 0.91 |
| TICS9 | 1–5 | 1 | 5 | 3.28 ± 0.96 |
| TICS10 | 1–5 | 1 | 5 | 3.26 ± 0.99 |
| TICS11 | 1–5 | 1 | 5 | 3.94 ± 0.76 |
| TICS12 | 1–5 | 2 | 5 | 3.93 ± 0.79 |
| TICS13 | 1–5 | 2 | 5 | 3.84 ± 0.75 |
| TICS14 | 1–5 | 1 | 5 | 3.97 ± 0.80 |
| TICS15 | 1–5 | 1 | 5 | 4.07 ± 0.75 |

TICS = the Three-dimensional Inventory of Character Strengths; CS = Character Strengths

**Table S2.** Prevalence of psychological distress, anxiety, and depression by demographic and clinical characteristics（n = 271）

| Characteristics | n(%) | DT≥4  n(%) | HADS-A≥10  n(%) | HADS-D≥7  n(%) |
| --- | --- | --- | --- | --- |
| **Age group (years)** |  |  |  |  |
| 15–24 | 20 (7.38) | 11 (55.00) | 7 (35.00) | 7 (35.00) |
| 25–29 | 22 (8.12) | 11 (50.00) | 5 (22.73) | 9 (40.91) |
| 30–34 | 59 (21.77) | 30 (50.85) | 11 (18.64) | 16 (27.12) |
| ≥35 | 170 (62.73) | 98 (57.65) | 42 (24.71) | 55 (32.35) |
| **Marital status** |  |  |  |  |
| Single | 51 (18.82) | 27 (52.94) | 12 (23.53) | 18 (35.29) |
| Married | 186 (68.63) | 102 (54.84) | 44 (23.66) | 57 (30.65) |
| Divorced/Widowed | 34 (12.55) | 21 (61.76) | 9 (26.47) | 12 (35.29) |
| **Fertility status** |  |  |  |  |
| Parous | 199 (73.43) | 109 (54.77) | 45 (22.61) | 62 (31.16) |
| Nulliparous | 72 (26.57) | 41 (56.94) | 20 (27.78) | 25 (34.72) |
| **Education** |  |  |  |  |
| Junior high school or below | 82 (30.26) | 46 (56.10) | 22 (26.83) | 26 (31.71) |
| Senior high school or vocational secondary school | 81 (29.89) | 45 (55.56) | 22 (27.16) | 27 (33.33) |
| Junior college diploma | 50 (18.45) | 28 (56.00) | 10 (20.00) | 20 (40.00) |
| Undergraduate or above | 58 (21.40) | 31 (53.45) | 11 (18.97) | 14 (24.14) |
| **Employment status** |  |  |  |  |
| Employed | 118 (43.54) | 63 (53.39) | 25 (21.19) | 32 (27.12) |
| Unemployed | 153 (56.46) | 87 (56.86) | 40 (26.14) | 55 (35.95) |
| **Dissatisfied with current financial status** |  |  |  |  |
| Strongly disagree | 32 (11.81) | 16 (50.00) | 4 (12.50) | 12 (37.50) |
| Disagree | 59 (21.77) | 28 (47.46) | 6 (10.17) | 10 (16.95) |
| Neutral | 108 (39.85) | 58 (53.70) | 35 (32.41) | 39 (36.11) |
| Agree | 52 (19.19) | 35 (67.31) | 14 (26.92) | 23 (44.23) |
| Strongly agree | 20 (7.38) | 13 (65.00) | 6 (30.00) | 3 (15.00) |
| **Insurance status** |  |  |  |  |
| No | 36 (13.28) | 29 (80.56) | 6 (16.67) | 10 (27.78) |
| Yes | 235 (86.72) | 121 (51.49) | 59 (25.11) | 77 (32.77) |
| **Residence** |  |  |  |  |
| Urban | 170 (62.73) | 95 (55.88) | 38 (22.35) | 51 (30.00) |
| Rural | 101 (37.27) | 55 (54.46) | 27 (26.73) | 36 (35.64) |
| **Time since diagnosis** |  |  |  |  |
| <1 month | 81 (29.89) | 48 (59.26) | 26 (32.10) | 31 (38.27) |
| 1–3 months | 89 (32.84) | 49 (55.06) | 17 (19.10) | 25 (28.09) |
| 3 months–1 year | 50 (18.45) | 20 (40.00) | 7 (14.00) | 10 (20.00) |
| 1–3 years | 26 (9.59) | 15 (57.69) | 7 (26.92) | 10 (38.46) |
| >3 years | 25 (9.23) | 18 (72.00) | 8 (32.00) | 11 (44.00) |
| **Disease stage** |  |  |  |  |
| I | 95 (35.06) | 47 (49.47) | 19 (20.00) | 26 (27.37) |
| II | 44 (16.24) | 26 (59.09) | 11 (25.00) | 14 (31.82) |
| III | 42 (15.50) | 26 (61.90) | 12 (28.57) | 16 (38.10) |
| IV | 28 (10.33) | 17 (60.71) | 7 (25.00) | 11 (39.29) |
| Uncertain | 62 (22.88) | 34 (54.84) | 16 (25.81) | 20 (32.26) |
| **Therapeutic methodᵃ** |  |  |  |  |
| Surgery |  |  |  |  |
| No | 57 (21.03) | 32 (56.14) | 15 (26.32) | 20 (35.09) |
| Yes | 214 (78.97) | 118 (55.14) | 50 (23.36) | 67 (31.31) |
| Chemotherapy |  |  |  |  |
| No | 117 (43.17) | 66 (56.41) | 33 (28.21) | 41 (35.04) |
| Yes | 154 (56.83) | 84 (54.55) | 32 (20.78) | 46 (29.87) |
| Targeted / Immunotherapy / Hormone therapy |  |  |  |  |
| No | 241 (88.93) | 130 (53.94) | 55 (22.82) | 69 (28.63) |
| Yes | 30 (11.07) | 20 (66.67) | 10 (33.33) | 18 (60.00) |
| Others |  |  |  |  |
| No | 259 (95.57) | 143 (55.21) | 59 (22.78) | 82 (31.66) |
| Yes | 12 (4.43) | 7 (58.33) | 6 (50.00) | 5 (41.67) |
| **Cancer type** |  |  |  |  |
| Cervical | 122 (45.02) | 67 (54.92) | 35 (28.69) | 39 (31.97) |
| Endometrial | 36 (13.28) | 18 (50.00) | 7 (19.44) | 11 (30.56) |
| Ovarian | 105 (38.75) | 58 (55.24) | 23 (21.90) | 36 (34.29) |
| Other | 8 (2.95) | 7 (87.50) | 0 (0.00) | 1 (12.50) |
| **Family relationship satisfaction** |  |  |  |  |
| Dissatisfied | 16 (5.90) | 11 (68.75) | 7 (43.75) | 7 (43.75) |
| Neutral | 29 (10.70) | 19 (65.52) | 9 (31.03) | 14 (48.28) |
| Satisfied | 112 (41.33) | 62 (55.36) | 28 (25.00) | 39 (34.82) |
| Very satisfied | 114 (42.07) | 58 (50.88) | 21 (18.42) | 27 (23.68) |
| **Disease understanding** |  |  |  |  |
| Unfamiliar | 29 (10.70) | 18 (62.07) | 7 (24.14) | 9 (31.03) |
| Neutral | 52 (19.19) | 32 (61.54) | 18 (34.62) | 19 (36.54) |
| Familiar | 144 (53.14) | 81 (56.25) | 33 (22.92) | 52 (36.11) |
| Very familiar | 46 (16.97) | 19 (41.30) | 7 (15.22) | 7 (15.22) |
| **Family history of cancer** |  |  |  |  |
| Yes | 32 (11.81) | 20 (62.50) | 7 (21.88) | 12 (37.50) |
| No | 239 (88.19) | 130 (54.39) | 58 (24.27) | 75 (31.38) |
| DT = Distress thermometer.  ^a^: The total number of participants counted in the “Therapeutic method” section exceeded the overall study population (n = 271), as some participants may have undergone combined therapy with two or more therapeutic methods. | | | | |

**Table S3.** Covariate-adjusted sensitivity analysis of the Gaussian graphical model

| Section | Indicator / Edge / Node | Primary network | Covariate-adjusted network | Change / correlation | Interpretation |
| --- | --- | --- | --- | --- | --- |
| Overall network similarity | Sample size | 271 | 271 | — | No sample loss after adjustment |
| Overall network similarity | Edge-weight Pearson correlation | — | — | 0.995 | Very high edge-weight similarity |
| Overall network similarity | Edge-weight Spearman correlation | — | — | 0.942 | Highly stable edge-weight ranking |
| Overall network similarity | Strength centrality Spearman correlation | — | — | 0.973 | Highly stable strength centrality ranking |
| Overall network similarity | Bridge strength Spearman correlation | — | — | 0.989 | Highly stable bridge strength ranking |
| Key cross-community edge | Curiosity–Distress | 0.000 | 0.000 | 0.000 | Not retained in either network |
| Key cross-community edge | Curiosity–Anxiety | −0.034 | −0.023 | +0.011 | Retained; same negative direction |
| Key cross-community edge | Curiosity–Depression | −0.078 | −0.065 | +0.013 | Retained; same negative direction |
| Key cross-community edge | Zest–Distress | −0.112 | −0.109 | +0.003 | Retained; same negative direction |
| Key cross-community edge | Zest–Anxiety | 0.000 | 0.000 | 0.000 | Not retained in either network |
| Key cross-community edge | Zest–Depression | 0.000 | 0.000 | 0.000 | Not retained in either network |
| Key strength centrality | Leadership | 1.119 | 1.070 | Rank 1 → 1 | Remained the highest strength node |
| Key strength centrality | Creativity | 1.052 | 1.030 | Rank 2 → 2 | Remained highly central |
| Key strength centrality | Curiosity | 1.016 | 0.949 | Rank 3 → 4 | Remained among the highly central nodes |
| Key strength centrality | Zest | 0.665 | 0.616 | Rank 16 → 16 | Low global strength but stable |
| Key bridge strength | Distress | 0.148 | 0.148 | Rank 1 → 2 | Remained a major bridge symptom |
| Key bridge strength | Depression | 0.136 | 0.152 | Rank 2 → 1 | Remained a major bridge symptom |
| Key bridge strength | Curiosity | 0.112 | 0.088 | Rank 3 → 4 | Remained a key bridge strength |
| Key bridge strength | Zest | 0.112 | 0.109 | Rank 4 → 3 | Remained a key bridge strength |

**Note.** The covariate-adjusted network was estimated after adjusting all network nodes for financial dissatisfaction, time since diagnosis, family relationship satisfaction, and disease understanding. Curiosity refers to TICS6, Zest to TICS10, Leadership to TICS12, and Creativity to TICS8. Edge weights are regularized partial correlations. Negative edge weights indicate inverse associations. Rank changes are shown as primary network rank → adjusted network rank. Bayesian network and in silico intervention analyses were not repeated in this sensitivity analysis because they were exploratory supplementary analyses and were interpreted as assumption-dependent probabilistic and simulation-based findings.

**Table S4.** Stable directed edges in the theory-constrained Bayesian network

| **No.** | **From** | **To** | **Strength** | **Direction** |
| --- | --- | --- | --- | --- |
| 1 | TICS7 | TICS6 | 1.000 | 0.694 |
| 2 | TICS8 | TICS9 | 1.000 | 0.679 |
| 3 | HADS-A | DT | 1.000 | 0.662 |
| 4 | HADS-D | HADS-A | 1.000 | 0.579 |
| 5 | TICS12 | TICS11 | 1.000 | 0.561 |
| 6 | TICS2 | TICS3 | 1.000 | 0.507 |
| 7 | TICS14 | TICS15 | 0.998 | 0.539 |
| 8 | TICS12 | TICS13 | 0.997 | 0.657 |
| 9 | TICS8 | TICS7 | 0.997 | 0.573 |
| 10 | TICS3 | TICS4 | 0.962 | 0.750 |
| 11 | TICS5 | TICS1 | 0.956 | 0.542 |
| 12 | TICS11 | TICS13 | 0.809 | 0.567 |
| 13 | TICS2 | TICS1 | 0.808 | 0.708 |
| 14 | TICS13 | TICS14 | 0.762 | 0.612 |
| 15 | TICS10 | TICS9 | 0.762 | 0.571 |
| 16 | TICS13 | TICS6 | 0.711 | 0.659 |
| 17 | TICS7 | TICS15 | 0.696 | 0.520 |
| 18 | TICS12 | TICS14 | 0.693 | 0.709 |
| 19 | TICS10 | DT | 0.645 | 1.000 |
| 20 | TICS11 | TICS15 | 0.639 | 0.671 |
| 21 | TICS13 | TICS15 | 0.616 | 0.607 |
| 22 | HADS-D | DT | 0.576 | 0.745 |
| 23 | TICS5 | TICS15 | 0.574 | 0.611 |
| 24 | TICS6 | HADS-D | 0.572 | 1.000 |
| 25 | TICS7 | TICS10 | 0.571 | 0.602 |
| 26 | TICS2 | TICS5 | 0.566 | 0.691 |
| 27 | TICS6 | TICS5 | 0.561 | 0.607 |
| 28 | TICS10 | TICS4 | 0.547 | 0.620 |
| 29 | TICS8 | TICS5 | 0.528 | 0.608 |
| 30 | TICS9 | TICS6 | 0.518 | 0.643 |
| 31 | TICS5 | TICS4 | 0.512 | 0.538 |

**Note.** Strength indicates the proportion of bootstrap resamples in which the directed edge is present; Direction indicates the probability of the displayed direction conditional on the edge being present.

**Table S5.** In-degree and out-degree characteristics of all nodes in the theory-constrained Bayesian network

| **Node** | **Character strength / symptom** | **In-degree** | **Out-degree** |
| --- | --- | --- | --- |
| TICS2 | Prudence | 0 | 3 |
| TICS8 | Creativity | 0 | 3 |
| TICS12 | Leadership | 0 | 3 |
| TICS7 | Humor | 1 | 3 |
| TICS10 | Zest | 1 | 3 |
| TICS11 | Fairness | 1 | 2 |
| HADS-D | Depression | 1 | 2 |
| TICS3 | Judgment | 1 | 1 |
| HADS-A | Anxiety | 1 | 1 |
| TICS13 | Teamwork | 2 | 3 |
| TICS9 | Social Intelligence | 2 | 1 |
| TICS14 | Honesty | 2 | 1 |
| TICS1 | Self-Regulation | 2 | 0 |
| TICS5 | Love of Learning | 3 | 3 |
| TICS6 | Curiosity | 3 | 2 |
| DT | Psychological distress | 3 | 0 |
| TICS4 | Perseverance | 3 | 0 |
| TICS15 | Kindness | 5 | 0 |

**Table S6.** Stable directed edges in the unconstrained Bayesian network

| **No.** | **From** | **To** | **Strength** | **Direction** |
| --- | --- | --- | --- | --- |
| 1 | HADS-A | DT | 1.000 | 0.685 |
| 2 | TICS7 | TICS6 | 1.000 | 0.679 |
| 3 | TICS8 | TICS9 | 1.000 | 0.673 |
| 4 | HADS-D | HADS-A | 1.000 | 0.573 |
| 5 | TICS12 | TICS11 | 1.000 | 0.558 |
| 6 | TICS2 | TICS3 | 1.000 | 0.504 |
| 7 | TICS12 | TICS13 | 0.998 | 0.660 |
| 8 | TICS14 | TICS15 | 0.998 | 0.538 |
| 9 | TICS8 | TICS7 | 0.996 | 0.566 |
| 10 | TICS3 | TICS4 | 0.964 | 0.751 |
| 11 | TICS5 | TICS1 | 0.955 | 0.546 |
| 12 | TICS6 | TICS10 | 0.810 | 0.732 |
| 13 | TICS11 | TICS13 | 0.809 | 0.574 |
| 14 | TICS2 | TICS1 | 0.804 | 0.721 |
| 15 | TICS13 | TICS14 | 0.764 | 0.613 |
| 16 | DT | TICS10 | 0.738 | 0.633 |
| 17 | TICS13 | TICS6 | 0.707 | 0.659 |
| 18 | TICS7 | TICS15 | 0.700 | 0.511 |
| 19 | TICS12 | TICS14 | 0.689 | 0.711 |
| 20 | TICS11 | TICS15 | 0.637 | 0.672 |
| 21 | TICS6 | HADS-D | 0.624 | 0.672 |
| 22 | TICS13 | TICS15 | 0.624 | 0.599 |
| 23 | TICS2 | TICS5 | 0.574 | 0.672 |
| 24 | TICS5 | TICS15 | 0.566 | 0.609 |
| 25 | TICS6 | TICS5 | 0.564 | 0.611 |
| 26 | HADS-D | DT | 0.561 | 0.711 |
| 27 | TICS7 | TICS10 | 0.554 | 0.650 |
| 28 | TICS10 | TICS4 | 0.553 | 0.583 |
| 29 | TICS9 | TICS6 | 0.528 | 0.637 |
| 30 | TICS8 | TICS5 | 0.523 | 0.608 |
| 31 | TICS1 | TICS10 | 0.523 | 0.593 |

**Note.** Strength indicates the proportion of bootstrap resamples in which the directed edge is present; Direction indicates the probability of the displayed direction conditional on the edge being present.

**Table S7.** Model-implied changes in psychological outcomes following hypothetical 1-SD increases in character-strength nodes

| **Simulated CS node** | **Overall model-implied change** | **Model-implied distress change (SD)** | **Model-implied anxiety change (SD)** | **Model-implied depression change (SD)** |
| --- | --- | --- | --- | --- |
| TICS6 | −0.230 | −0.167 | −0.218 | −0.306 |
| TICS7 | −0.110 | −0.091 | −0.097 | −0.144 |
| TICS8 | −0.078 | −0.081 | −0.050 | −0.104 |
| TICS11 | −0.033 | −0.015 | −0.036 | −0.046 |
| TICS10 | −0.032 | −0.123 | 0.013 | 0.016 |
| TICS12 | −0.031 | −0.035 | −0.027 | −0.030 |
| TICS13 | −0.028 | −0.026 | −0.017 | −0.040 |
| TICS3 | −0.025 | −0.017 | −0.041 | −0.017 |
| TICS9 | −0.024 | −0.013 | −0.023 | −0.036 |
| TICS1 | −0.016 | −0.001 | −0.026 | −0.022 |
| TICS15 | −0.015 | −0.027 | −0.017 | −0.000 |
| TICS14 | −0.004 | −0.002 | 0.006 | −0.016 |
| TICS5 | 0.005 | −0.010 | −0.001 | 0.026 |
| TICS4 | 0.011 | 0.017 | 0.005 | 0.010 |
| TICS2 | 0.018 | 0.017 | 0.011 | 0.026 |

**Note.** Values are standardized differences from the model-implied baseline. Negative values denote lower model-implied scores. These conditional simulation results are not estimates of causal intervention efficacy or clinical treatment effects.
