# Supplementary material for: Character strengths, psychological distress, anxiety, and depression in adolescents and young adults with gynecologic cancer: A network analysis
Source: Asia Pac J Oncol Nurs. 2026 Sep 10;13:101027. doi: 10.1016/j.apjon.2026.101027 (PMC13634094; doi:10.1016/j.apjon.2026.101027)
Supplement: Supplementary file 3 [file mmc2.doc]

STROBE Statement—Checklist of items that should be included in reports of ***cross-sectional studies***

|  | Item No | Recommendation | **Location in manuscript** |
| --- | --- | --- | --- |
| **Title and abstract** | 1 | (*a*) Indicate the study’s design with a commonly used term in the title or the abstract | Page 1 / Line 3 |
|  |  | (*b*) Provide in the abstract an informative and balanced summary of what was done and what was found | Page 1 / Line 29-53 |
| Introduction | | |  |
| Background/rationale | 2 | Explain the scientific background and rationale for the investigation being reported | Page 2 / Line 59-124 |
| Objectives | 3 | State specific objectives, including any prespecified hypotheses | Page 3 / Line 125-142 |
| Methods | | |  |
| Study design | 4 | Present key elements of study design early in the paper | Page 4 / Line 146 |
| Setting | 5 | Describe the setting, locations, and relevant dates, including periods of recruitment, exposure, follow-up, and data collection | Page 4 / Line 146-147 |
| Participants | 6 | (*a*) Give the eligibility criteria, and the sources and methods of selection of participants | Page 4 / Line 149-154 |
| Variables | 7 | Clearly define all outcomes, exposures, predictors, potential confounders, and effect modifiers. Give diagnostic criteria, if applicable | Page 5 / Line 209-255 |
| Data sources/ measurement | 8* | For each variable of interest, give sources of data and details of methods of assessment (measurement). Describe comparability of assessment methods if there is more than one group | Page 5 / Line 189-207 |
| Bias | 9 | Describe any efforts to address potential sources of bias | Page 5 / Line 189-207 |
| Study size | 10 | Explain how the study size was arrived at | Page 4 / Line 156-187 |
| Quantitative variables | 11 | Explain how quantitative variables were handled in the analyses. If applicable, describe which groupings were chosen and why | Page 5 / Line 213-218 |
| Statistical methods | 12 | (*a*) Describe all statistical methods, including those used to control for confounding | Page 6 / Line 257-347 |
|  |  | (*b*) Describe any methods used to examine subgroups and interactions | Page 6 / Line 261-262 |
|  |  | (*c*) Explain how missing data were addressed | Page 5 / Line 203-207 |
|  |  | (*d*) If applicable, describe analytical methods taking account of sampling strategy | Page 4 / Line 175-178 |
|  |  | (*e*) Describe any sensitivity analyses | Page 7 / Line 316-321 |
| Results | | |  |
| Participants | 13* | (a) Report numbers of individuals at each stage of study—eg numbers potentially eligible, examined for eligibility, confirmed eligible, included in the study, completing follow-up, and analysed | Page 4 / Line 183-187 |
|  |  | (b) Give reasons for non-participation at each stage | Page 4 / Line 182-183 |
|  |  | (c) Consider use of a flow diagram | No flow diagram included |
| Descriptive data | 14* | (a) Give characteristics of study participants (eg demographic, clinical, social) and information on exposures and potential confounders | Page 8 / Line 359-363  Table 2 |
|  |  | (b) Indicate number of participants with missing data for each variable of interest | Page 4 / Line 182-183 |
| Outcome data | 15* | Report numbers of outcome events or summary measures | Page 9 / Line 368-370  Tables S1 and S2 |
| Main results | 16 | (*a*) Give unadjusted estimates and, if applicable, confounder-adjusted estimates and their precision (eg, 95% confidence interval). Make clear which confounders were adjusted for and why they were included | Page 9 / Line 373-463 |
|  |  | (*b*) Report category boundaries when continuous variables were categorized | Page 5 / Line 223-255 |
|  |  | (*c*) If relevant, consider translating estimates of relative risk into absolute risk for a meaningful time period | Not applicable |
| Other analyses | 17 | Report other analyses done—eg analyses of subgroups and interactions, and sensitivity analyses | Page 9 / Line 405-414  Page 10 / Line 435-449 |
| Discussion | | |  |
| Key results | 18 | Summarise key results with reference to study objectives | Page 11 / Line 467-481 |
| Limitations | 19 | Discuss limitations of the study, taking into account sources of potential bias or imprecision. Discuss both direction and magnitude of any potential bias | Page 13 / Line 592-614 |
| Interpretation | 20 | Give a cautious overall interpretation of results considering objectives, limitations, multiplicity of analyses, results from similar studies, and other relevant evidence | Page 11 / Line 482-557 |
| Generalisability | 21 | Discuss the generalisability (external validity) of the study results | Page 13 / Line 559-582 |
| Other information | | |  |
| Funding | 22 | Give the source of funding and the role of the funders for the present study and, if applicable, for the original study on which the present article is based | Page 15 / Line 654-657 |

*Give information separately for exposed and unexposed groups.

**Note:** An Explanation and Elaboration article discusses each checklist item and gives methodological background and published examples of transparent reporting. The STROBE checklist is best used in conjunction with this article (freely available on the Web sites of PLoS Medicine at http://www.plosmedicine.org/, Annals of Internal Medicine at http://www.annals.org/, and Epidemiology at http://www.epidem.com/). Information on the STROBE Initiative is available at www.strobe-statement.org.
